# Biomarkers of Atrial Fibrillation Recurrence in Patients with Paroxysmal or Persistent Atrial Fibrillation Following External Direct Current Electrical Cardioversion

**DOI:** 10.3390/biomedicines11051452

**Published:** 2023-05-16

**Authors:** Ozan Demirel, Alexander E. Berezin, Moritz Mirna, Elke Boxhammer, Sarah X. Gharibeh, Uta C. Hoppe, Michael Lichtenauer

**Affiliations:** 1 Department of Internal Medicine II, Division of Cardiology, Paracelsus Medical University Salzburg, 5020 Salzburg, Austria; o.demirel@salk.at (O.D.); m.mirna@salk.at (M.M.); e.boxhammer@salk.at (E.B.); s.gharibeh@salk.at (S.X.G.); u.hoppe@salk.at (U.C.H.); m.lichtenauer@salk.at (M.L.); 2 Internal Medicine Department, Zaporozhye State Medical University, 69035 Zaporozhye, Ukraine

**Keywords:** atrial fibrillation, electrical cardioversion, post-procedural complications, biomarkers

## Abstract

Atrial fibrillation (AF) is associated with atrial remodeling, cardiac dysfunction, and poor clinical outcomes. External direct current electrical cardioversion is a well-developed urgent treatment strategy for patients presenting with recent-onset AF. However, there is a lack of accurate predictive serum biomarkers to identify the risks of AF relapse after electrical cardioversion. We reviewed the currently available data and interpreted the findings of several studies revealing biomarkers for crucial elements in the pathogenesis of AF and affecting cardiac remodeling, fibrosis, inflammation, endothelial dysfunction, oxidative stress, adipose tissue dysfunction, myopathy, and mitochondrial dysfunction. Although there is ample strong evidence that elevated levels of numerous biomarkers (such as natriuretic peptides, C-reactive protein, galectin-3, soluble suppressor tumorigenicity-2, fibroblast growth factor-23, turn-over collagen biomarkers, growth differential factor-15) are associated with AF occurrence, the data obtained in clinical studies seem to be controversial in terms of their predictive ability for post-cardioversion outcomes. Novel circulating biomarkers are needed to elucidate the modality of this approach compared with conventional predictive tools. Conclusions: Biomarker-based strategies for predicting events after AF treatment require extensive investigation in the future, especially in the presence of different gender and variable comorbidity profiles. Perhaps, a multiple biomarker approach exerts more utilization for patients with different forms of AF than single biomarker use.

## 1. Introduction

Atrial fibrillation (AF) is the most common form of sustained cardiac arrhythmia in the world [1]. The prevalence of AF advances with increasing age. After the age of 80, atrial fibrillation affects 10–17% of the population [2]. The morbidity is increased and mortality rises up to 3.5-fold in men and women [3]. Along with it, AF frequently occurs in patients at higher risk of cardiovascular diseases (CVD) as well as among individuals with known CVD [4]. Unfortunately, AF and CVD exacerbate each other and mutually intervene in prognosis. Indeed, patients with any form of AF demonstrated poorer clinical outcomes if there is concomitant heart failure (HF), coronary artery disease (CAD), type 2 diabetes mellitus (T2DM), obesity, obstructive sleep apnea, chronic kidney disease (CKD), or peripheral artery disease [5,6,7]. Further, the prognosis of patients with AF is poorer than the prognosis of patients with various CVD and comorbid conditions (i.e., HF, CKD) without AF [8]. Multi-morbidity among patients with AF seems to play a pivotal role in natural evolution of primary and secondary AF through direct and indirect impact on the structural and/or electrophysiological abnormalities that occur in AF [9,10]. AF influences electrical remodeling, i.e., shortening of refractoriness due to the high atrial rate itself, resulting in adverse cardiac remodeling [11]. Yet, the persistence of AF itself modulates the risk of cerebrovascular and cardiovascular events [12,13].

The management of AF includes either rhythm restoration or rate control along with comorbidity management, prevention of stroke, and systemic thromboembolism [14]. Synchronized electrical cardioversion can terminate AF. Combined with sedation, it is a safe procedure and highly effective, restoring sinus rhythm in more than 90% [15,16,17]. It is important to detect AF recurrence after successful electrical cardioversion. In this case, early cardioversion could prolong the subsequent duration of sinus rhythm and slow disease progression compared to delayed sinus rhythm restoration [18].

Although the current clinical protocol of initial AF management seems to be very useful in practice [1], it poses challenges in predicting incidental AF and early detection of AF-related complications [19,20]. There are many factors associated with AF recurrence, such as duration of AF, higher age, sex, HF, LA volume index, chronic obstructive pulmonary disease, hypertension, obstructive sleep apnea, hyperthyroidism, smoking, and obesity [21,22]. However, the role of biomarkers reflecting the different stages of AF pathogenesis has not been completely understood. The purpose of the study is to summarize the current evidence on the value of various biomarkers in predicting the likelihood of AF recurrence after electrical cardioversion.

## 2. Promoting Factors and Electrophysiological/Anatomical Substrates of AF

**Figure 1:**
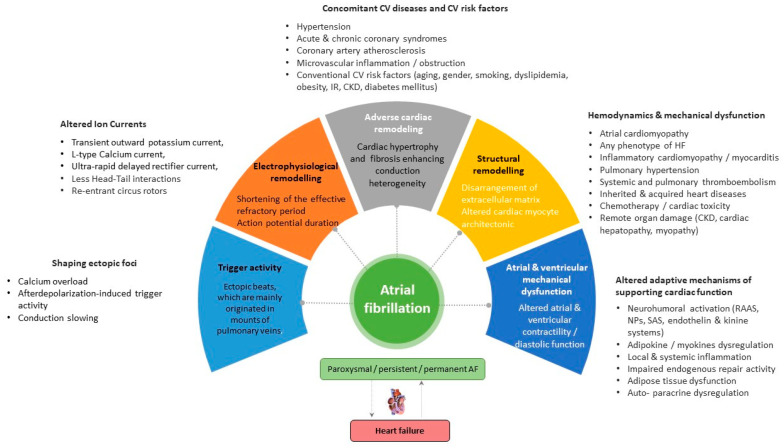
Promoting factors and plausible pathogenetic mechanisms of AF. Abbreviations: AF, atrial fibrillation; CV, cardiovascular; CKD, chronic kidney disease; SAS, sympathoadrenal system; IR, insulin resistance; HF, heart failure; RAAS, renin-angiotensin-aldosterone system.

Vulnerable substrates for the occurrence, support, and recurrence of AF are electrophysiological and adverse cardiac remodeling, along with structural remodeling, mechanical dysfunction, and trigger activity, which are mediated by genetic ion channel alterations, concomitant cardiovascular (CV) diseases (acute and chronic coronary syndromes, multifocal atherosclerosis, primary and secondary cardiomyopathy, etc.), CV risk factors (hypertension, smoking, obesity, diabetes mellitus, resistance to insulin, and dyslipidemia), and comorbidities (chronic obstructive pulmonary disease, bronchial asthma, chronic kidney disease) (Figure 1). In addition, concomitant hemodynamic factors as a result of numerous diseases (heart failure, atrial cardiomyopathy, pulmonary hypertension, inherited and acquired heart diseases, myocarditis) and conditions (chemotherapy, cardiac toxicity) play a crucial role in secondary structural remodeling of the heart [23,24,25]. These factors contribute to AF occurrence by maintaining afterdepolarization-induced triggered ectopic activity, focal enhanced automaticity, altered function of ion channels, micro-reentrant circus rotor, less dynamic head–tail interactions during re-entry in cardiac tissue, altered ion accumulation on the dynamics of re-entry and electrical heterogeneity [26]. Indeed, head–tail interactions have previously been known to have a causative impact on the dynamics of the reentrant action potential, which plays a pivotal role in inducing AF [26]. To note, intracellular ions, mainly Ca^2+^ and Na^+^, accumulated during reentrant arrhythmia through the rapid repetitive cellular excitation may lead to spontaneous termination of re-entry or break-up of the re-entry loop into multiple pathways resulting in AF. Along with it, the initiation and persistence of AF are controlled by both parasympathetic and sympathetic stimulation, as well as hormonal influences, which also seem to play a role in AF recurrence [27]. However, the continuous interaction between electrophysiological, structural, and anatomical remodeling leads to intercellular uncoupling and a pro-fibrotic response, which is crucial for trigger activity, the presence of AF, and the transformation of cardiac dysfunction into HF [28].

### 2.1. Electrophysiological Remodeling

Electrophysiological remodeling affects variable changes in specific ionic currents, such as a reduction in transient outward potassium current, L-type calcium current, and ultra-rapid delayed rectifier current, as well as shortening of the effective refractory period and prolongation of the action potential, which are also associated with the increase in the stimulation rate [29,30]. The overload of intracellular calcium in cardiac myocytes and its spontaneous release from the sarcoplasmic reticulum seems to be a major factor in the occurrence of delayed afterdepolarizations and triggered ectopic activity in the myocardium [30]. Although sympathetic activation and direct stimulation by angiotensin-II are classic mechanisms of enhancing propensity for AF, there are numerous other mechanisms that intervene in altered afterdepolarizations. They mainly include a reduced inward rectifier, as well as increased activity of the Na/Ca exchanger and residual beta-adrenergic responsiveness [31,32]. Yet, alterations in the regulation and accumulation of intracellular calcium can be a result of properly persistent AF and alternative arrhythmogenic mechanisms (intramural decremented conduction, transmural heterogeneity of repolarization, prolongation of QT-interval, and block of the premature impulse), which are activated due to progress of pre-exciding CV diseases including HF and coronary artery syndromes [33]. To note, asynchronous down-regulation of voltage-dependent potassium currents and L-type calcium currents between layers of myocardium through the calcium/calmodulin-dependent kinase II signal pathway activated by hemodynamic factors (fluid overload, hypertension), ischemia/hypoxia, hormonal dysfunction (hyperthyroidism), perivascular edema due to microvascular inflammation, impaired mitochondrial metabolism and oxidative stress due to metabolic diseases/conditions (diabetes mellitus, obesity, myopathy, insulin resistance) and cardiac hypertrophy may support electrophysiological remodeling [34,35,36,37,38,39]. Although the role of hemodynamic factors and ischemia in shaping AF risk is well established [34,35], the impact of metabolic influences on electrophysiological remodeling is not always obvious. For instance, among patients with thyroid dysfunction, free thyroxine levels but not thyroid-stimulating hormone concentrations are associated with an increased risk of incident AF regardless of preexisting CV disease [37]. On the other hand, a hypothyroid state may directly induce myocardial fibrosis via stimulating autophagy and inhibiting TGF-β1/Smad2 signal transduction pathway [38]. Obesity and T2DM link glycemic fluctuations to electrophysiological remodeling that leads to the onset and maintenance of AF through mitochondrial dysfunction, oxidative stress, and inflammation [39]. Chan YH et al. (2019) [40] reported that insulin resistance (IR) was associated with significantly increased sarcoplasmic reticulum calcium content and diastolic calcium sparks in the atrial myocardium. Moreover, IR increased collagen accumulation and superoxide production in the atrial myocardium through increased synthesis of transforming growth factor beta 1 (TGF-β1) and abnormal upregulation of calcium-homeostasis-related proteins, such as oxidized CaMKIIδ, phosphorylated-phospholamban, phosphorylated-RyR-2, and sodium–calcium exchanger [40].

Yet, subcellular mechanisms underlying electrophysiological remodeling seem to relate to the alteration of connexin 43 expression, which is a principal ventricular gap junction protein [40,41]. However, significant changes in connexin 43 phosphorylation were found to be more closely associated with timing AF persistence and the presence of HF [42]. In particular, these changes can even explain an association of such powerful components of electrophysiological features as increased transmural dispersion in refractoriness and conduction with increased inducibility of AF and low efficacy of electrical cardioversion [43,44]. Moreover, this may be a novel paradigm of electrophysiological remodeling based on the timing of conduction abnormalities in connection to dynamic changes in connexin 43 isoforms, cardiac dysfunction, and comorbidities [43,44]. Indeed, there is strong evidence of the fact that apelin-13-an aliphatic multifunctional peptide, mainly originated from the myocardium, skeletal muscles, and liver-increased connexin 43 through autophagy inhibition and inducing AKT and mTOR phosphorylation and thereby decreases susceptibility to cardiac arrhythmias including AF and cardiomyocyte death [45,46]. Yang M et al. (2022) [47] recently reported that the apelin/AMPK/mTOR signaling pathway, which regulates angiotensin II-mediated autophagy and apoptosis of cardiac myocytes, is under close control of miRNA-122-5p. The overexpression of miRNA-122-5p leads to exacerbation of cardiac and vascular hypertrophy, cardiac fibrosis, and dysfunction. Thus, the overexpression of miRNA-122-5p may be an underlying mechanism of binding myocardial fibroblasts and activation of AF. On the other hand, angiotensin-II acts as a promoter of expression of pro-apoptotic molecules, such as P62 and Bax, and as a mediator of mTOR phosphorylation, which downregulates LC3II, beclin-1, and contributes to the imbalance of autophagy and apoptosis in the myocardium. These changes were associated with increased myocardial accumulation of collagen I and collagen III, overexpression of TGF-beta-1 and connective tissue growth factor (CTGF), as well as downregulation of myocardial expression of apelin, angiotensin-converting enzyme-2 (ACE2), and growth differential factor-15 (GDF-15) [47,48,49]. These facts confirm a close interplay between the Apelin-APJ axis and ACE2-GDF-15-porimin signaling in angiotensin-II-mediated myocardial hypertrophy and fibrosis, which are crucial substrates for AF occurrence and prolongation. Therefore, they modulate the relationship between electrophysiological and anatomical cardiac remodeling.

### 2.2. Adverse Cardiac Remodeling

**Figure 2:**
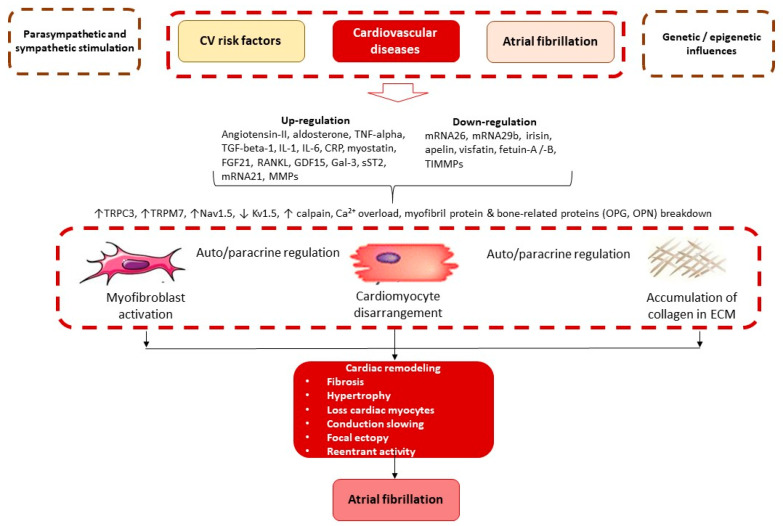
Molecular mechanisms of AF. Abbreviations: CV, cardiovascular; CRP, C-reactive protein; ECM, extracellular matrix; FGF21, fibroblast growth factor-21; GDF15, growth differential factor-15; Gal-3, galectine-3; TGF-beta-1 transforming growth factor beta-1, TNFα tumor necrosis factor alpha; TRPC3 transient receptor potential channel-3; TRPM7, transient receptor potential cation channel, subfamily M, member 7; Nav1.5, voltage-gated sodium channel, Kv1.5, voltage-gated potassium channel; MMPs. Matrix metalloproteinases; sST2, soluble suppression of tumorigenesis-2; RANKL, tumor necrosis factor ligand superfamily member 11; OPG, osteoprotegerin.

Adverse cardiac remodeling in AF patients includes AF-related atrial remodeling and cardiac remodeling due to concomitant CV diseases [50]. Both variants may be associated with sinus node dysfunction, variability in conduction gaps due to parasympathetic/sympathetic stimulation and epigenetic regulation of intercellular communication, cardiac cell-to-cell heterogeneity, and extracellular matrix alteration [50,51,52,53]. Therefore, the overlap between both variants of remodeling is mediated by concomitant hemodynamic changes such as valvular regurgitation [54]. AF-related alteration of atrial structure starts with the differentiation of cardiac fibroblasts into myofibroblasts, which is regulated by numerous triggers, including angiotensin-II, noradrenaline, thyroid hormones, inflammatory cytokines, chemokines, matrix metalloproteinases, galectine-3, soluble suppression of tumorigenesis-2 (sST2), TGF-beta-1 and microRNAs (Figure 2).

These triggers contribute to the altered expression of several ion channel proteins, such as transient receptor potential channel-3 (TRPM3) and member 7 TRPC3, which regulate intracellular calcium flow, and mediate dysfunction of the ion channels on the surfaces of target cells [54]. Angiotensin-II, aldosterone, endothelin, and catecholamines, as well as several inflammatory cytokines (TNF-alpha, interleukin-2) acting as signaling molecules contribute to fibroblast proliferation via Ca^2+^ entry via transient receptor potential channels (voltage-gated sodium [Nav1.5] and potassium channels [Kv1.5]) [55]. In addition, myofibril protein breakdown is stimulated by overexpressed calpain, which is activated by intracellular Ca^2+^ loading [56]. As a result, activated myofibroblasts not only produce several types of collagens shaping collagen deposition and cardiac fibrosis but also directly interact with cardiomyocytes promoting AF [57]. Moreover, myofibroblasts and fibrotic areas interfere with atrial tissue conduction and lead to intercellular uncoupling. Indeed, interactions between myofibroblast and cardiomyocyte alter conduction and elicit focal activity in the atria [58]. Finally, cell uncoupling, along with cardiac myocyte disarmament and extensive fibrosis, lead to gap junctions and non-uniformity of anisotropy modulating AF. In addition, pre-exceeding CV diseases, such as myocardial infarction, cardiomyopathies, myocarditis, and cardiac hypertrophy, through the strength of local mechanical forces, loss of cardiac myocytes and extensive fibrosis intervene in anisotropy modality of cardiac electrical conductivity and shaping arrhythmogenic substrate [59].

Although collagen accumulation in the myocardium is regulated by autocrine/paracrine and neurohumoral mechanisms, the atria are more prone to extracellular matrix remodeling and collagen deposition than the ventricles. Possibly, it depends on the distinguished presence of matrix metalloproteinases, their inhibitors, and pro-inflammatory molecules involved in the subsequent regulation of collagen synthesis and degradation. The accumulation of collagen, other matrix proteins (elastin, fibronectin 1, fibrillin 1), and proteoglycans in abundance lead to severe heterogeneous areas in the atria with variable alteration of electrophysiological properties [59]. This eventually leads to changes in myocardial cell architecture, such as elongation and disturbed alignment of demarcated fibers. This subsequently causes anisotropic changes in the entire myocardium, mediating a discrepancy between transverse and longitudinal electrical conduction leading to AF.

## 3. Electrical Cardioversion of AF: Safety and Outcomes

It seems that standard external direct current electrical cardioversion is a well-developed urgent treatment strategy for patients presenting recent-onset AF [1,60]. Numerous retrospective one-center studies and multicenter trials yielded 86–88% efficacy of the approach in restoring sinus rhythm along with 6–10% relapse of AF in a short-term perspective (7–28 days) [61,62,63]. Overall, electrical cardioversion in AF patients who required emergency department transportation was associated with infrequent hospital admission and few mild-to-moderate complications [61]. However, the duration of AF in the majority of studies was less than 48 h in 99% of the patients. Burton JH et al. (2004) [61] observed in a retrospective multicenter study that electrical cardioversion had an 86% success rate, and only 10% of the patients returned to the emergency department within 7 days. Fried AM et al. (2021) [16] reported that the efficacy of this procedure, defined as restoration of sinus rhythm, reached 88% in routine clinical practice, whereas major complications (post-cardioversion stroke, thromboembolic events, jaw thrust maneuver for hypoxia, and overnight observation for hypotension) and predefined minor adverse events (frequently related to general anesthesia, skin burns) were detected in 0.3% and 14%, respectively. In addition, electrical cardioversion was about 2.5 times more effective than conventional pharmacological treatment in restoring sinus rhythm [62,63]. Although there are numerous potential complications of electrical cardioversion (i.e., ventricular fibrillation, thromboembolism due to inadequate anticoagulant therapy, nonsustained ventricular tachycardia, various forms of atrial arrhythmias, bradycardia, transient left bundle branch block, myocardial necrosis, asymptomatic myocardial dysfunction, acute HF, transient hypotension, pulmonary edema, and stroke), they occur less frequently than recurrent AF. Further, 6.4% of patients revisited the emergency department within 30 days, and 4.8% returned with AF or atrial flutter. It is noteworthy that the return visit rate for patients with relapsed AF varies between 3% and 17% [64].

Overall, 30-day all-cause mortality among AF patients undergoing direct-current electrical cardioversion was 0.8% [65]. Data received from the FIRE (Atrial Fibrillation/flutter Italian Registry) registry showed that predictors of unsuccessful electrical cardioversion were onset of AF > 48 h, concomitant HF, increasing age, syncope, transient ischemic attack (TIA)/stroke as well as previous admission to a non-cardiology department [66]. The investigators also found several predictors of in-hospital mortality in this patient population, including age, HF, diabetes mellitus, previous admission to a non-cardiology department, and TIA/stroke [66]. Thus, patients at low risk for thromboembolic complications, including stroke and heart failure, seem to benefit more from electric cardioversion than other individuals with recent-onset AF [67].

Another reason for physicians to use this approach may be cost savings and a short period of emergency department admission [67,68]. Houghton AR et al. (2000) [69] and Boriani G. et al. (2007) [70] did not identify concise hemodynamic predictors of successful external electrical cardioversion or relapses after electrical cardioversion among patients with persistent AF or atrial flutter. However, only two predictors (duration of arrhythmia ≥1 year and previous cardioversion) were found to be powerful for this matter [69,70], whereas, in previous investigations, relapse of AF was associated with reduced left ventricular ejection fraction [71]. Along with it, standard external biphasic direct current electrical cardioversion has better efficacy than monophasic electrical cardioversion (360-J) for restoration of sinus rhythm in AF patients, although dual external monophasic 360-J cardioversion may increase the success rate as a rescue technique after failing standard external direct current cardioversion [72,73]. In this concept, the prediction of plausible cardiovascular events, including relapsed AF, with a biomarker strategy seems promising in patients with recent-onset AF.

## 4. Predictors for AF Recurrence Following Electrical Cardioversion

Biomarkers reflecting the complex pathophysiological mechanisms underlying AF seem to be an effective tool to predict rhythm status after cardioversion as well as other AF-related complications, which can intervene in mortality, hospital admission, cardiovascular (CV), and non-CV outcomes (Table A1).

### 4.1. Natriuretic Peptides

Natriuretic peptides (brain natriuretic peptide [BNP], N-terminal pro-B-type natriuretic peptide (NT-proBNP), mid-regional pro-A type natriuretic peptide (MR-proANP)) serve as circulating cardiac biomarkers of biomechanical stress, adverse cardiac remodeling and fluid overload with established diagnostic and predictive values for acute and chronic HF involving any phenotypes [74,75]. Along with it, elevated levels of NPs were strongly associated with all-cause and CV mortality and urgent hospitalization among patients with AF, T2DM, CKD, hypertension, and cardiac hypertrophy [76,77]. Moreover, NT-proBNP and BNP were found to be predictors for AF [78,79,80]. However, it has been suggested that restoration of sinus rhythm through effective electric cardioversion may associate with a reduction in NP concentrations and thereby predict the recurrence of new episodes of arrhythmia. Xu X et al. (2017) [81] observed in a meta-analysis that low levels of BNP and NT-proBNP were associated with the maintenance of sinus rhythm and that the baseline concentrations of both biomarkers may be a predictor of AF recurrence after successful electrical cardioversion. Ari H. et al. (2008) [82] reported that a significant decrease in BNP levels 30 min after electric cardioversion corresponded to six-month maintenance of sinus rhythm in follow-up.

In the GAPP-AF (The gene expression patterns for the prediction of atrial fibrillation) study, Meyre PB (2022) [83] investigated 21 conventional and new circulating biomarkers reflecting inflammation, myocardial injury, cardiac biomechanical stress, and renal dysfunction before and 30 days after electrical cardioversion and evaluated plausible associations of changes in circulating biomarker levels with rhythm status at 30-day follow-up. The patients included in the study had no acute HF, severe valvular disease, or life-limiting active or chronic serious concomitant diseases. The authors found that low levels of NT-proBNP were independently associated with sinus rhythm restoration after electric cardioversion. On the other hand, initial levels of BNP and NT-proBNP in patients with persistent AF without established CVD did not predict long-term sinus rhythm maintenance, although conversion to sinus rhythm related to a significant decrease in circulating BNP but not NT-proBNP level [84]. In contrast, NT-proBNP levels were found to be a predictor of AF recurrence 30 days after successful electric cardioversion among patients with persistent AF and CV risk factors, including hypertension and dyslipidemia [85]. In another study, pre-procedural NT-proBNP levels, but not post-procedural levels of the peptide, independently predicted the relapse of AF after successful electrical cardioversion [86]. These controversial issues perhaps may relate to the presence of concomitant HF. Indeed, in the CAPRAF (Candesartan in the Prevention of Relapsing Atrial Fibrillation) trial, plasma NT-proBNP concentrations measured before electrical cardioversion did not predict cardioversion success nor the relapse of AF in patients without HF [87]. Mabuchi N et al. (2000) [88] noticed that low atrial natriuretic peptide (ANP) and high BNP levels before electric cardioversion were independent predictors of recurrent AF in mild chronic HF patients. Moreover, the authors established that ANP to BNP ratio <0.44 was a significant risk factor for AF recurrence [88]. The BNP level of 700 fmol/mL or higher on day 7 after cardioversion was most predictive for AF recurrence (sensitivity, 78%; specificity, 71%), whereas ANP did not predict the relapse of AF [89]. Buccelletti F. et al. (2011) [90] measured the levels of NT-proBNP in 200 patients admitted to the emergency department due to new-onset AF (<2 weeks) regardless of HF presence. The authors found that NT-proBNP levels of either ≤450 pg/mL or >1800 pg/mL seem to show positive and negative predictive values for cardioversion in rate-control and rhythm-control strategies, respectively. In the range of 450 to 1800 pg/mL, NT-proBNP did not exhibit serious clinical utility [90]. However, it remained unclear whether continuous monitoring of the dynamic changes of NPs after sinus rhythm restoration predicts recurrent AF [91]. Overall, the restoration of sinus rhythm after electric cardioversion in AF patients is associated with a decrease in circulating levels of NPs and low levels of NT-proBNP predicts a sustainable maintains of sinus rhythm in follow-up.

### 4.2. Biomarkers of Fibrosis

Cardiac fibrosis was found to be closely associated with AF. Circulating biomarkers of fibrosis have already been proposed as a promising tool in its evaluation, but which biomarkers are most appropriate for AF remains unclear [92]. There are a large number of circulating biomarkers, which characterize the accumulation of extracellular matrix components and fibrosis, such as soluble suppressor tumorigenicity-2 (sST2), galectin-3 (Gal-3), procollagen type III N terminal peptide (PIIINP), type I collagen carboxyl telopeptide (ICTP), and fibroblast growth factor 23 (FGF-23) [93].

#### 4.2.1. Galectin-3

Gal-3 is a multifunctional galactose-binding protein that belongs to the transforming growth factor beta superfamily and a biomarker of fibrosis, involved in atrial remodeling, cardiac fibrosis, and AF [94]. Previous studies revealed that patients with AF had higher Gal-3 values than non-AF patients, regardless of their comorbidity profile [95,96]. Moreover, elevated Gal-3 levels were independently associated with paroxysmal non-valvular AF [97].

There is ample evidence of a close relation between elevated Gal-3 levels, atrial remodeling (i.e., parameters of left atrial dimension, volume, compliance, and contractility) and AF recurrence following successful electrical cardioversion [98,99,100]. Gürses KM et al. (2019) [98] reported that pre-cardioversion Gal-3 levels in persistent AF corresponded to a higher left atrial volume index and were associated with early AF recurrence following successful sinus rhythm restoration. In contrast, Cichoń M et al. (2021) [101] did not find any link between circulating Gal-3 levels and the risk of recurrent AF in obese and non-obese patients with persistent AF. The same results were obtained in another study involving 75 non-HF patients with paroxysmal or persistent AF referred for electrical cardioversion [102]. Although the authors of the study established a correlation between the Gal-3 levels and oxidative stress and inflammation in AF patients, only circulating myeloperoxidase, but not Gal-3, was associated with the maintenance of sinus rhythm in a multivariate model, possibly due to the small number of patients and relatively early stage of AF [102]. Whether these changes may be explained in connection with single nuclear polymorphisms of the Gal-3 gene has not been fully elucidated [103]. Thus, the predictive ability of Gal-3 for sinus rhythm restoration following successful electrical cardioversion requires thorough investigations in face-to-face comparison with other biomarkers before implementation in clinical practice.

#### 4.2.2. sST2

Soluble suppression of Tumorigenicity 2 protein (sST2) is part of the interleukin 1 receptor/Toll-like superfamily, which is related to cardiac inflammation, fibrosis, and also remodeling. Current clinical guidelines for HF consider sST2 as an alternative biomarker of all-cause and CV mortality as well as HF-related complications, including hospital admission, especially in HF with preserved ejection fraction (HFpEF) [74,75]. Although sST2 is involved in cardiac fibrosis, local and systemic inflammation, and atrial and ventricular remodeling, its role in predicting clinical outcomes of electrical cardioversion of AF remains uncertain [104,105]. In patients with HF and acute myocardial infarction, elevated sST2 levels were a powerful risk factor for new-onset AF [106,107]. Moreover, in AF patients without concomitant cardiovascular disease, sST2 concentrations were positively associated with LV myocardial strain and T1 mapping indices [108]. Previous studies have demonstrated significant predictability of AF recurrence after cryoballoon and radiofrequency ablation using sST2 [109,110,111].

It appears that limited evidence exists regarding a discriminatory effect of sST2 measured before and after electrical cardioversion on AF recurrence. Wałek P. et al. (2020) [112] found that sST2, but not Gal-3, predicted sinus rhythm maintenance after successful electrical cardioversion of AF in patients without HF. Perhaps, sST2 may be considered as part of a multimarker panel for the prediction of AF recurrence along with NPs and Gal-3. Overall, sST2 seems to be a promising predictive biomarker for AF recurrence after electrical cardioversion, cryoballoon, and radiofrequency ablation.

#### 4.2.3. Other Biomarkers of Fibrosis

Begg GA (2017) [113] investigated an association of biomarkers related to fibrosis and collagen metabolism with procedural risk and AF recurrence rates among 79 patients undergoing external direct current cardioversion in comparison with 40 age-and-disease-matched volunteers. The authors found that Gal-3, PIIINP, and ICTP were not predictive for AF recurrence after electrical cardioversion, whereas FGF-23 had a weak predictive ability for relapsing AF [113]. In contrast, Kawamura M. et al. (2012) [114] found no discriminatory levels of interleukin-6, high-sensitivity C-reactive protein, BNP, renin, and aldosterone for the 24-month recurrence rate of AF, whereas baseline serum levels of PIIINP > 0.72 U/mL predicted AF relapse. Thus, there is a serious discrepancy between biomarker levels corresponding to the presence of atrial fibrosis confirmed by cardiac magnetic resonance imaging and their discriminatory properties for recurrent AF [115,116,117].

Furthermore, elevated serum levels of FGF-23 strongly correlated with the total number of major cardiovascular events and left atrial dimension in paroxysmal AF patients as well as with new-onset AF in sinus rhythm patients presenting CV risk factors, but not with the maintenance of sinus rhythm during follow-up [117,118,119]. Meta-analysis of 15 clinical studies, enrolling 36,017 participants, revealed that elevated serum FGF-23 levels, but not GDF15 levels, were associated with the risk of AF [120]. A meta-analysis of 15 clinical trials involving 36,017 participants found that elevated serum FGF-23 levels, but not GDF15 levels, were associated with AF risk [120]. However, it remains unclear whether these results also apply to patients undergoing electrical cardioversion.

### 4.3. Biomarkers of Inflammation

#### 4.3.1. GDF15

GDF15 is a member of the TGF-beta superfamily whose expression is increased in response to biomechanical myocardial stress, inflammation, or ischemia/hypoxia [121]. GDF15 is involved in the regulation of energy homeostasis, thermogenesis, and eating behavior [122]. Yet, GDF15 also exerts anti-inflammatory and anti-proliferative properties, although the underlying molecular mechanisms are still unclear [123]. Elevated GDF15 levels were found in patients with any phenotypes of chronic HF, stroke, AF, and T2DM [124,125,126,127,128]. In the general population, GDF-15 did not show a positive association with the prevalence of AF and the risk of AF occurrence [129]. The suitability of GDF15 for predicting bleeding and/or atrial thrombosis during anticoagulant therapy remains questionable [130]. Clinical evidence for the discriminative value of GDF15 for AF relapse or sinus rhythm maintenance is extremely limited. There is one small study that prospectively included 82 patients with persistent AF [101]. Although log10 serum GDF-15 levels correlated positively with the CHA2DS2-VASc score, there was no close association between GDF-15 levels and sinus rhythm maintenance in patients after successful electric cardioversion [101]. Thus, a discriminative potency of GDF15 for the prediction of clinical efficacy of electrical cardioversion among patients with nonvalvular/valvular AF is not completely understood and requires scrutiny in large clinical studies.

#### 4.3.2. hs-CRP

High-sensitivity C-reactive protein (hs-CRP) is a classic biomarker of inflammation and is a component of the inflammatory profile observed in AF patients. Elevated hs-CRP levels were found in patients with all forms of nonvalvular/valvular AF, regardless of etiology and concomitant comorbidities [131,132]. hs-CRP predicted new-onset AF both in the general population as well as in patients with established cardiovascular or metabolic diseases, such as HF, acute myocardial infarction, T2DM, and metabolic syndrome [133,134,135]. Among patients with AF complicated by systemic thromboembolism, the levels of hs-CRP correlated positively with the CHA2DS2-VASc score [136].

Loricchio ML et al. (2007) [137] investigated plausible predictors for a 1-year risk of AF recurrence after electrical cardioversion. In a Cox regression analysis, the authors found that age, gender, hypertension, T2DM, LVEF, left atrial diameter, use of various antiarrhythmic and antihypertensive (including angiotensin-converting enzyme inhibitors or angiotensin II antagonists) drugs, and statins were not associated with relapsing AF. On the contrary, a low quartile of hs-CRP levels was found to be a strong predictor for this outcome [137]. Lombardi F. et al. (2008) [138] did not find any changes in hs-CRP levels after cardioversion in patients with persistent AF and preserved LVEF, regardless of the post-procedural underlying rhythm. However, NT-proBNP levels decreased significantly in patients who maintained sinus rhythm but not in those who had AF. Yet, baseline hs-CRP levels, but not echocardiographic features of atrial dysfunction and initial NT-proBNP levels, predicted recurrences of AF after cardioversion in patients without pre-existing left ventricular dysfunction [138]. Barassi A et al. (2012) [139] and Korantzopoulos P et al. (2008) [140] confirmed that in patients with persistent AF and preserved LVEF, elevated hs-CRP levels independently predicted subacute AF recurrence rate, whereas NT-proBNP concentrations were not associated with arrhythmic outcome but corresponded to the alterations of cardiac hemodynamics secondary to the presence of AF. 

Overall, there is ample strong evidence that elevated preprocedural hs-CRP levels may provide independent predictive information for both successful electrical cardioversion of AF and maintenance of sinus rhythm after conversion [141,142]. The meta-analysis by Liu et al. [143], which included six prospective observational studies (n = 366 patients), showed that peripheral blood CRP levels were higher in patients with failed electric cardioversion than in those with successful restoration of sinus rhythm. In another meta-analysis by Yo CH et al. (2014) [144], a cut-off value of 1.9 mg/L hs-CRP predicted long-term AF recurrence (77% sensitivity, 65% specificity), and more than 3 mg/L predicted short-term AF relapse (73% sensitivity, 71% specificity). Thus, the measurement of CRP levels before the procedure may provide additional prognostic information about the success of sinus rhythm maintenance.

In addition, there are data illustrating that hs-CRP levels measured shortly after electrical cardioversion may be a powerful biomarker for assessing the risk of relapsing AF in the long-term. In particular, Celebi OO et al. (2011) [145] reported that hs-CRP levels measured before and 2 days after electrical cardioversion predicted the 1-year risk of AF relapse. Whether postprocedural hs-CRP provides more information to predict the event than preprocedural hs-CRP is still unclear. However, elevated levels of hs-CRP predicted new-onset AF in the general population and among patients with known cardiovascular diseases, while their role as a marker of sustainable sinus rhythm control places under question.

### 4.4. Myokines and Adipocytokines

Several interdependent canonic signaling pathways, such as the renin-angiotensin-aldosterone system; TGF-beta pathway, inflammatory chemokines, and cytokines lead to cardiac fibrosis through modulation of oxidative stress and inflammation. However, the direct mechanical stretch may act as a modulator of extracellular matrix remodeling by attenuating the expression of matrix metalloproteinases and their inhibitors. Recently, another signaling pathway has been identified that induces atrial fibrosis via the secretion of adipokines from epicardial, perivascular, and adipose tissue white adipocytes. In addition, recent studies have shown that myokines derived from cardiac and skeletal muscle myocytes may act as adaptive regulators of extracellular matrix remodeling and can attenuate fibrosis [146,147]. Depending on their origin, adipokines and myokines may modulate myofibroblast capabilities, regulate myocyte energy homeostasis and protect against inflammation and fibrosis [148,149]. However, some pro-fibrotic adipokines and myokines can switch a generation of reactive oxygen species to pro-inflammatory and pro-fibrotic stimuli, stimulate myofibroblast differentiation through JAK/STAT3 and JNK/c-Jun signaling, interfere with myocyte electrophysiology, and promote fibrosis in the myocardium [150,151,152]. Numerous previous studies have shown that resistin, apelin, and adiponectin are adipokines associated with several known risk factors for AF and risk of AF [153,154,155,156]. A recent meta-analysis of 34 studies (total number of patients = 31,479) showed that some adipokines, mainly adiponectin, apelin, and resistin, were associated with the risk of AF in the pooled univariate data, whereas the associations were not apparent after multivariate adjustment [157]. However, there is limited evidence of the relation between adipokine and myokine signatures and the risk of AF-related outcomes after electric cardioversion.

#### 4.4.1. Apelin

Apelin is a multifunctional regulatory peptide with potential cytoprotective properties. It is a ligand of the angiotensin II protein J receptor (APJ) receptor and belongs to the G protein-coupled receptor family [158]. Apelin mRNA is widely expressed in tissues such as the cardiovascular, central nervous, adipose, skeletal muscles, and gastrointestinal systems. The Apelin/APJ axis mediates signal transduction for regulating energy homeostasis, including glucose and lipid metabolism, mitochondrial function, angiogenesis, cellular proliferation, and differentiation [159]. Furthermore, apelin inhibits apoptosis, decreases myocardial infarction size, and prevents myocardial ischemia/reperfusion injury via the PI3K/Akt and ERK1/2 caspase signaling. It is also engaged in the autophagy pathway, attenuation of inflammatory reactions, and prevention of atherosclerotic plaque formation [160]. Several controversial issues remain regarding whether the apelin/APJ system is essential for regulating atrial and ventricular remodeling by alleviating myocardial hypertrophy induced by angiotensin II, oxidative stress, and TGF-beta1 [161,162,163]. Nevertheless, it has been shown that atrial wall stretching can activate the myocardial APJ axis [164]. Moreover, APJ was found to be essential for stretch-induced contractility and may also induce ectopic electrical activity by Ca^2+^ sensitization of myofilaments. It is believed that apelin counteracts APJ’s stretch-triggered hypertrophy signaling by suppressing Ca^2+^ transients [164]. Along with it, there are a variety of vascular effects of apelin that include regulation of systolic and diastolic blood pressure through vasorelaxation and an increase in regional blood flow [165,166].

Previous studies have shown that circulating levels of apelin were sufficiently lower in patients with established cardiovascular diseases (coronary artery disease, myocardial infarction, acute coronary syndrome, HF), T2DM, and obesity than in healthy volunteers [167,168]. A meta-analysis of 30 studies revealed a negative association of apelin serum levels with cardiovascular diseases [169]. However, peripheral blood apelin concentrations were not only significantly decreased in AF patients compared with healthy controls but also independently predicted recurrent AF in patients with persistent AF. This included cases occurring after pulmonary vein isolation in subjects without structural heart disease [170,171,172]. It has been suggested that low apelin levels may interfere with AF susceptibility through elevated atrial NADPH-dependent oxidative stress and the TGF-β/Smad2/α-SMA pathway associated with mitochondrial dysfunction and myocardial fibrosis [173,174]. In addition, the apelin/APJ axis might be involved in atrial thrombus formation among AF patients, possibly as a result of concomitant downstream plasminogen activator inhibitor-1 (PAI-1) [175].

The predictive role of apelin for AF occurrence after electric cardioversion remains uncertain. In a small comparative study, Kallergis EM et al. (2010) [176] showed that baseline apelin levels did not independently predict AF recurrence, whereas NT-proBNP did. Interestingly, maintenance of sinus rhythm after electrical cardioversion resulted in an increase in serum apelin levels and a decrease in serum NT-pro-BNP levels. However, more studies are needed to clarify apelin’s discriminative potency for AF recurrence in AF patients after electrical cardioversion, with comparisons of apelin’s predictive value to other conventional and promising biomarkers.

#### 4.4.2. Irisin

Irisin was previously described as a hormone-like myokine, which is mainly secreted by skeletal muscle and myocardium and is a derivative of the membrane protein fibronectin type III domain-containing 5 (FNDC5) [177]. Exercise increases serum levels of irisin, which exert cytoprotective effects on remote organs and tissues, including the heart, kidney, vasculature, bones, and brain [177,178]. Irisin interacts with αV/β5 integrin on the surface of target cells and induces a wide range of biological effects, including stimulation of glucose and lipid metabolism, increase in insulin resistance, browning of visceral adipose tissue, thermogenesis, angiogenesis, survival of osteoblasts, and production of bone-related proteins such as sclerostin [179,180,181,182].

Serum irisin levels were significantly decreased in obese and T2DM patients compared with nondiabetic controls, as well as in patients with known cardiovascular disease (cardiac hypertrophy, stable coronary artery disease, chronic HF, multifocal atherosclerosis) compared with healthy volunteers [183,184]. On the contrary, acute HF, acute coronary syndrome, and acute myocardial infarction were associated with an increase in irisin levels, which is considered an adaptive factor that reduces endothelial damage by inhibiting inflammatory reactions and suppressing oxidative stress [185,186,187]. A low irisin level was described as an independent predictor of clinical outcomes in HF patients [188,189]. Although patients with HFpEF and AF had significantly lower irisin levels than those without AF [190], the role of irisin in predicting AF-related events, including relapse after electric cardioversion, has not yet been investigated.

#### 4.4.3. Bone-Related Proteins

There is growing strong evidence that inflammatory responses are involved in the development of AF and its complications. Bone-related proteins are matricellular peptides that mediate diverse biological functions and are involved in many pathological conditions in cardiovascular disease, including fibrosis, microvascular inflammation, calcification, extracellular remodeling, and atherosclerotic plaque formation [191]. Bone-related proteins, such as osteoprotegerin (OPG) and TNF-related apoptosis-inducing ligand (TRAIL), mediate a link between cardiovascular comorbidities and diseases, such as diabetes mellitus, CKD, atherosclerosis, HF, vascular calcification, and the occurrence of AF [192]. Indeed, cardiovascular comorbidities were associated with higher OPG levels and lower TRAIL levels immediately after the first hours of AF paroxysm [125,193]. Furthermore, osteopontine (OPN) levels were related to an increased risk of systemic thromboembolism and ischemic stroke in patients with AF [194]. OPG and OPN were found to be predictors of HF outcomes independent of AF presence and have been included in a multiple-scoring system to predict survival in chronic HF [195]. In a small clinical study involving 100 non-CVD patients with and without AF recurrence, low levels of bone morphogenetic protein 10 exhibited predictive value for sinus rhythm maintenance with a striking similarity to NT-proBNP [83]. However, it remains unclear whether these biomarkers have prognostic abilities for the maintenance of sinus rhythm in AF patients after electrical cardioversion.

### 4.5. Biomarkers of Oxidative Stress and Endothelial Dysfunction

#### 4.5.1. Cell-Free Circulating DNA

Cell-free circulating DNA (cfcDNA) circulates in two main pools: circular and single-stranded molecules belonging to mitochondrial-derived and nuclear-derived subpopulations, reflecting patterns of DNA methylation and a variety of neutrophil extracellular traps (NETosis) [196,197]. The cfcDNA are determined in subdetectable concentrations under certain physiological conditions, such as physical exercise, whereas increased circulating levels of these fragments are strongly associated with cardiovascular, autoimmune, rheumatic diseases, infections, and malignancy [198,199,200,201,202]. The main causes of cfcDNA production are mitochondrial dysfunction and inflammation, which are powerful drivers of numerous diseases and conditions, including AF [203]. 

Wiersma M. et al. (2020) [204] reported that levels of cell-free circulating mitochondrial DNA (cfc-mtDNA) were significantly increased in patients with paroxysmal AF undergoing AF treatment, especially in men and in patients with AF recurrence after electrical cardioversion or pulmonary vein isolation. In contrast, cfc-mtDNA levels gradually decreased in patients with persistent AF and long-standing persistent AF. Nevertheless, the authors suggested that cfc-mtDNA levels might be associated with the stage of AF and the risk of AF recurrence after treatment, especially in men. Gender differences in descriptive values of cfc-mtDNA for AF recurrence remain poorly understood but could be related to different comorbidities in both subpopulations. However, another study found no significant changes in mtDNA copy number in the peripheral blood of AF patients of different sex and age [205]. Perhaps, cfcDNA may be included in the multiple biomarker models with the aim of improving their predictive potency in AF patients with low levels of NT-proBNP or in AF patients with malignancy who are treated with chemotherapy. 

#### 4.5.2. mRNA

MicroRNAs (miRNAs) participate in atrial remodeling and cardiac fibrosis, contributing to the development of AF [206]. Garcia-Elias A et al. (2021) [207] established that circulating levels of miR-199a-5p and miR-22-5p, which regulate fibrogenic response in the myocardium, were higher in HFrEF patients with AF than in those without AF [207]. MiR-21, which corresponds to atrial fibrosis, is associated with the risk of persistent AF in patients with left atrial enlargement [208]. Interestingly, increased circulating levels of miR-1-3p, which is a myosine gene regulator involved in hypertrophy, myocardial infarction, and cardiac arrhythmogenesis, predicted a high risk of subclinical AF [209]. MiR423, which downregulates fibrosis-related genes such as collagen I, collagen III, fibronectin, and TGF-beta, may be a pivotal factor in stratifying patients at risk of AF occurrence and persistence [210]. Moreover, differences in miRNA expression in the atrial myocardium of men and women may mediate a sex-specific association between circulating miRNAs in plasma and AF at the population level [206]. In addition, there is evidence that epigenetic regulation of NETosis may participate in the development of AF susceptibility. As a matter of fact, miR-146a and miR21 may provide prognostic information in patients with AF [211,212] due to its direct effects on NETosis. In a study by da Silva AMG (2018) [213], miR-21, miR-133b, and miR-499, which are directly involved in the downregulation of apoptosis and fibrosis, were found to be directly involved in AF. However, it remains to be determined whether a signature of mi-Rs can be used to predict poor response to AF treatment, including electrical cardioversion. At the same time, Zhou Q et al. (2018) [213] reported that among 123 miRs affecting cardiac fibrosis, hypertrophy, and inflammation by relation with the SMAD7 and FASLG genes, only miR-21 demonstrated a positive correlation with left atrial low-voltage areas in patients with persistent AF and was associated with post-ablation outcome. Overall, the signature of miRs appears to be a more promising tool for higher AF risk than for outcomes after treatment, although this conjecture needs to be further investigated in the future.

#### 4.5.3. Asymmetric Dimethylarginine

Asymmetric dimethylarginine (ADMA) is a well-known biomarker of endothelial dysfunction that indirectly reflects vascular NO production and exhibits certain predictive information for mortality and morbidity of cardiovascular diseases, including AF [214,215]. In the population-based Gutenberg Health Study (n = 5000), ADMA levels were correlated with left ventricular hypertrophy and AF prevalence [216]. An ARISTOTLE (Apixaban for Reduction in Stroke and Other Thromboembolic Events in Atrial Fibrillation) substudy showed that elevated ADMA levels exhibited a weak association with thromboembolic events in AF patients treated with anticoagulants (warfarin or apixaban) for a median of 1.9 years [217]. The investigators found that tertile groups of ADMA levels were sufficiently associated with death, stroke, and systemic embolism and that incorporating ADMA into CHA2DS2-VASc or HAS-BLED predictive models significantly improved C-indices for those clinical outcomes [217].

There is strong evidence that acute and persistent episodes of AF seem to show elevated ADMA levels accompanied by increased biomarkers of ischemic myocardial injury like cardiac troponins [218]. In the animal AF model, ADMA concentrations in peripheral blood returned to normal within 24 h after successful electrical cardioversion [218]. Along with it, increased circulating levels of ADMA in AF may be reduced by a Mediterranean diet and statin treatment [219,220]. Thus, being closely associated with thrombus formation and CHADS2/CHA2DS2-VASc score, ADMA is a biomarker for predicting pro-thrombotic risk in AF [221,222].

There are controversial data for ADMA’s predictive ability regarding AF recurrence after electrical cardioversion. Xia W et al. (208) [223] reported that elevated ADMA levels were strongly associated with an increased risk of AF relapse within 1 month after electrical cardioversion. On the contrary, Tveit A et al. (2010) [224] found that the levels of ADMA and the L-arginine/ADMA ratio did not exert predictive ability for sinus rhythm maintenance after electrical cardioversion, while the L-arginine/ADMA ratio remained elevated in patients with sinus rhythm for 6 months compared with patients with AF recurrence. The discriminative potency of ADMA may be strongly related to comorbidities. Indeed, serum ADMA levels were not associated with incident AF in the general population after adjusting for other cardiovascular risk factors [224]. Overall, the utility of ADMA refines clinical risk stratification in AF regardless of the treatment strategy.

## 5. Conclusions

Previous clinical studies demonstrated limited ability to predict the efficacy of electrical cardioversion with conventional biomarkers, which described adverse cardiac remodeling, biomechanical stress, fibrosis, inflammation, endothelial dysfunction, oxidative stress, and mitochondrial dysfunction. Epigenetic biomarkers such as miRs and biomarkers of oxidative stress and inflammation such as cfcDNA appear to show highly variable results in predicting post-procedural events. A biomarker-based strategy for predicting events after AF treatment requires extensive future investigation, especially in different gender and variable comorbidity profiles. Therefore, a multiple biomarker approach may be more useful than using a single biomarker for patients with different forms of AF. Large clinical trials are needed to make direct face-to-face comparisons with different biomarkers and their combinations.

## Data Availability

Not applicable.

## Appendix A

**Table A1:** Predictive values of different biomarkers in prediction of AF-related complications after electrical cardioversion.

Biomarkers	Population	Observation Period	Significance/Outcomes	References
Biomechanical stress				
BNP	58 patients with persistent AF and preserved LVEF	6 months	Baseline BNP level and the magnitude of its decrease after successful cardioversion predicted AF recurrence	[82]
NT-proBNP and BMP10	100 non-CVD patients with and without AF recurrence	30-day follow-up	Low NT-proBNP levels and BMP10 levels after electric cardioversion predicted sinus rhythm restoration	[83]
BNP and NT-proBNP	43 patients with persistent AF	18 months	Pre- and post-procedural levels of BNP and NT-proBNP did not predict new episodes of AF	[84]
NT-proBNP	199 patients with persistent AF	30 days	The levels of NT-proBNP > 500 ng/L predicted recurrence of AF in 30 days after successful electrical cardioversion	[85]
NT-pro-BNP	40 patients with persistent AF	1 month	Elevated baseline NT-pro-BNP predicted AF recurrence after electric cardioversion	[86]
NT-pro-BNP	171 patients with persistent AF without HF	1 month	Pre-cardioversion and post-cardioversion NT-pro-BNP levels did not predict a relapse of AF in patients without HF	[87]
ANP and BNP	71 HF patients with persistent AF	1 month	Low ANP and high BNP levels before electric cardioversion independently predicted recurrent AF	[88]
ANP and BNP	60 patients with persistent AF	12 months	The BNP level ≥700 fmol/mL on day 7 after cardioversion predicted AF recurrence. ANP level was not predictive of AF recurrence	[89]
NT-proBNP	200 patients with newly onset AF with and without HF	1 month	NT-proBNP levels of either ≤450 pg/mL or >1800 pg/mL had positive and negative predictive values for cardioversion in rate-control and rhythm-control strategies	[90]
Cardiac fibrosis				
Gal-3	90 patients with persistent AF	3 months	Serum Gal-3 level independently predicted early AF recurrence following successful direct-current electrical cardioversion.	[98]
Gal-3	82 patients with persistent AF	1 month	Baseline serum levels of Gal-3 were not associated with a risk of recurrent AF	[101]
Gal-3	75 non-HF patients with paroxysmal or persistent AF	1 year	Pre-procedural Gal-3 levels did not predict recurrent AF	[102]
sST2	80 patients with persistent AF without HF	12 months	Serum levels of sST2 predict sinus rhythm maintenance after cardioversion of AF in patients without HF	[112]
FGF-23	79 patients with persistent AF	12 months	FGF-23, but not Gal-3, PIIINP, and ICTP, had weak predictive ability for relapsing AF	[113]
PIIINP	88 patients with maintenance of sinus rhythm and 54 patients with AF recurrence	24 months	Baseline PIIINP levels >0.72 U/mL independently predicted AF recurrence after electric cardioversion	[114]
Inflammation				
GDF15	82 patients with persistent AF	1 month	GDF-15 levels correlated positively with the CHA2DS2-VASc score, but not associated with a risk of recurrent AF after electric cardioversion	[101]
hs-CRP	102 patients with non-valvular persistent AF	1 year	Low levels of hs-CRP were associated with long-term maintenance of sinus rhythm after electrical cardioversion for AF	[137]
hs-CRP	53 patients with persistent AF and a mean LVEF of 58.7 ± 6%	3 weeks	No changes in hs-CRP levels and decrease in NT-proBNP levels after effective cardioversion. Pre-procedural levels of hs-CRP predicted recurrence rate of AF	[138]
hs-CRP	57 patients with a mean LVEF of 58.7 ± 6%	3 weeks	Pre-procedural levels of hs-CRP, but not NT-proBNP, predicted recurrence rate of AF	[139]
hs-CRP	60 patients who received amiodarone for sinus rhythm maintenance	3 years	Pre-procedural levels of CRP >0.43 mg/dL were an independent predictor of AF recurrence	[140]
hs-CRP	106 patients with a history of symptomatic AF lasting ≥ 1 day	36 days	Pre-procedural hs-CRP levels ≥0.06 mg/dL predicted both AF recurrence and maintenance of sinus rhythm	[141]
hs-CRP	56 patients with persistent AF	180 days	Pre-procedural hs-CRP <0.8 mg/L was significantly associated with lower AF recurrence rates and maintenance of sinus rhythm	[142]
hs-CRP	216 patients with persistent AF	12 months	The baseline and 2-day levels of hs-CRP levels contributed a risk of AF recurrence	[145]
Apelin and NT-proBNP	40 patients with persistent AF and 15 controls in sinus rhythm	1 month	Pre-procedural apelin levels were lower and NT-pro-BNP levels were higher in patients with AF compared to controls. Cardioversion led to an increase in apelin levels and a decrease in NT-proBNP levels. Apelin did not predict AF recurrence, but NT-proBNP did	[176]
Biomarkers of oxidative stress and mitochondrial dysfunction				
cfc-mtDNA	59 non-AF patients undergoing cardiac surgery, 100 patients with paroxysmal AF, 116 patients with persistent AF, 20 longstanding-persistent AF individuals and 84 control individuals	-	Elevated cfc-mtDNA levels were found in patients with paroxysmal AF undergoing electrical cardioversion or pulmonary vein isolation, as well as in patients with AF relapse after AF treatment. In patients with persistent AF and longstanding persistent AF, the levels of cfc-mtDNA gradually decreased	[204]
miR-199a-5p and miR-22-5p	49 HFrEF with AF and 49 HFrEF with sinus rhythm	-	Elevated levels of circulating miR-199a-5p and miR-22-5p were associated with AF in HFrEF patients	[207]
miR-21	60 persistent AF patients and 60 matched sinus rhythm volunteers	-	Circulating miR-21 positively correlates with the quantification of left atrial fibrosis and is associated with the risk of persistent AF in patients with left atrial enlargement	[208]
miR-1-3p	64 consecutive patients with cryptogenic stroke, 9 patients with AF and 9 individuals with sinus rhythm	6 and 12 months	Elevated plasma levels of miR-1-3p predicted AF	[209]
miR-21, miR-133a, miR-133b, miR-150, miR-328, and miR-499	5 acute new-onset AF patients, 16 well-controlled AF and 15 control	-	miR-21, miR-133b, and miR-499, which downregulate apoptosis and fibrosis, were found to be directly related to AF	[213]
Biomarkers of endothelial dysfunction				
ADMA	64 patients with persistent AF	1 month	High levels of ADMA were strongly associated with an increased risk of AF relapse after electrical cardioversion	[222]
ADMA	98 patients with persistent AF	6 months	Changes in ADMA did not predict rhythm outcome after electrical cardioversion	[223]

Abbreviations: ADMA, asymmetric dimethylarginine; ANP, atrial natriuretic peptide; CVD, cardiovascular disease; BNP, brain natriuretic peptide; BMP10, bone morphogenetic protein 10; HF, heart failure; hs-CRP, high-sensitivity C-reactive protein; LVEF, left ventricular ejection fraction; NT-proBNP, N-terminal pro-B-type natriuretic peptide; sST2, soluble suppressor tumorigenisity-2; Gal-3, galectin-3; PIIINP, procollagen type III N terminal peptide; ICTP, type I collagen carboxyl telopeptide; FGF-23, fibroblast growth factor 23, cfc-mtDNA, cell-free circulating mitochondrial DNA.
